# Exceptional Hydrogen Storage Performance of Ti-Decorated C_3_B_2_ Quantum Dot: A Comprehensive First-Principles Study

**DOI:** 10.3390/molecules31060960

**Published:** 2026-03-12

**Authors:** Seyfeddine Rahali, Ridha Ben Said, Youghourta Belhocine, Bakheit Mustafa

**Affiliations:** 1 Department of Chemistry, College of Science, Qassim University, Buraydah 51452, Saudi Arabia; r.said@qu.edu.sa; 2 Laboratory of Catalysis, Bioprocess and Environment, Department of Process Engineering, Faculty of Technology, University of 20 August 1955, Skikda 21000, Algeria; y.belhocine@univ-skikda.dz

**Keywords:** hydrogen storage, C_3_B_2_ quantum dot, density functional theory, Ti decoration, reversible capacity

## Abstract

The development of lightweight materials with high and reversible hydrogen storage capacity remains a key materials design challenge. Here, we investigate pristine and Ti-decorated C_3_B_2_ quantum dots using DFT, DLPNO-CCSD(T), and statistical thermodynamics. Pristine C_3_B_2_ strongly chemisorbs H_2_ (E_ads_ = −0.93 eV), while Ti decoration moderates the interaction to a reversible regime (E_ads_ = −0.39 eV) through a balanced Kubas-type mechanism. Structural analysis shows that the Ti center becomes saturated at approximately five H_2_ molecules via Kubas-type coordination, while additional hydrogen molecules are stabilized in the vicinity of the Ti–C_3_B_2_ framework through cooperative interactions. Sequential adsorption shows that up to 20 H_2_ molecules can be stored per Ti–C_3_B_2_ unit. Thermodynamic and kinetic analyses reveal moderate desorption temperatures (≈322–366 K) and ultrafast release times, ensuring efficient cycling. Under realistic operating conditions (30/3 atm; 298/373 K), Ti–C_3_B_2_ achieves a reversible capacity of 20.10 wt%, surpassing DOE targets. These results highlight Ti-decorated C_3_B_2_ quantum dots as a promising, design-tunable platform for next-generation solid-state hydrogen storage.

## 1. Introduction

The combustion of molecular hydrogen (H_2_) is a new sustainable and ecologically acceptable energy source [1]. Transporting and storing hydrogen remains a significant barrier for commercial purposes [2,3]. The traditional H_2_ storage methods, such as liquefaction, storage in metal hydrides, and solid-state absorption, are still ineffective due to safety, weight, and cost concerns [4]. Thus, in order to create the hydrogen economy, hydrogen storage must be considered in materials with high gravimetric/volumetric density and good adsorption/desorption capabilities [5]. In other words, for effective onboard applications, it is essential to store hydrogen at optimal pressures and temperatures, ensuring easy reversibility and rapid kinetics [6]. Consequently, there has been a surge of interest in the concept of solid-state hydrogen storage that employs the adsorption/desorption mechanism. To achieve an efficient and reversible hydrogen storage medium, it is recommended to have hydrogen adsorbed in molecular form, with a physisorption process, provided that the adsorption energy falls within the range of −0.10 to −0.60 eV/H_2_ [7]. The US Department of Energy (DOE) has established a target for the gravimetric density of H_2_ that is greater than 5.5 wt% at 298 K and 30 atm during adsorption, and at 373 K and 3 atm during desorption [8,9].

In parallel to adsorption-based nanostructured materials, conventional metal hydrides and complex hydrides have been extensively investigated for hydrogen storage [10]. For example, MgH_2_ exhibits a high theoretical gravimetric capacity of 7.6 wt%, but its practical application is hindered by high desorption temperatures and sluggish kinetics [11]. Complex hydrides such as alanates (e.g., NaAlH_4_) and borohydrides (e.g., LiBH_4_) can reach theoretical capacities exceeding 10 wt%; however, they often require elevated temperatures for hydrogen release and may suffer from limited reversibility or complex regeneration processes [12]. Recent reviews emphasize that achieving simultaneously high gravimetric capacity, moderate desorption temperatures, and rapid kinetics remains a major challenge in solid-state hydrogen storage materials [13,14]. In this context, lightweight adsorption-based systems modulated by transition-metal centers represent an alternative strategy aimed at combining high reversible capacity with near-ambient operating conditions.

A prospective storage material for H_2_ should exhibit a substantial surface area and a low molecular weight [15]. Materials comprising light elements would be an appropriate candidate for adsorbent substances [16,17]. Extensive exploration has been conducted on the potential of carbon-based, boron-based, and other materials capable of storing substantial quantities of hydrogen [18,19,20,21,22]. Indeed, several computational studies have been conducted on carbon-based structures in various forms for the purpose of hydrogen storage [23,24]. Examples include carbon foams, hydrogen-loaded carbon nanotubes, graphene, and graphane sheets [25,26]. All theoretical studies in the literature indicate that these pristine materials exhibit limited interaction with H_2_ molecules, leading to a low binding energy that is not conducive to reversible hydrogen storage [20]. Graphene exhibits a binding energy ranging from −0.04 eV/H_2_ to −0.10 eV/H_2_ for the storage of molecular hydrogen, attributed to its weak interaction [27].

In recent years, transition-metal decoration has emerged as an effective strategy to overcome the weak physisorption observed in pristine carbon-based materials. In particular, Ti-functionalized graphene, borophene, and hexagonal boron nitride (h-BN) nanosheets have demonstrated adsorption energies typically ranging from −0.30 to −0.60 eV/H_2_, enabling reversible molecular hydrogen storage under near-ambient conditions [28,29,30]. In these systems, the adsorption mechanism is commonly governed by Kubas-type interactions involving σ-donation from H_2_ to vacant metal d-orbitals and partial back-donation into the σ* antibonding orbital. Despite their favorable adsorption energetics, however, their gravimetric capacities generally remain limited (approximately 5–8 wt%) due to the relatively high atomic mass of extended two-dimensional substrates and the limited density of accessible metal sites.

Zero-dimensional nanocages such as Ti–C_24_ and Ti–B_12_N_12_ have also been proposed as promising hydrogen hosts [31,32]. The enhanced curvature and local polarization in these cage-like systems strengthen metal anchoring and can increase hydrogen uptake compared to planar materials. Nevertheless, adsorption energies in such systems often approach the upper bound of the reversible window, potentially hindering facile desorption under practical conditions. Similarly, porous coordination frameworks such as Ti–MOF-74 exhibit weaker adsorption energies (−0.15 to −0.25 eV/H_2_) and good reversibility [33], but their overall gravimetric performance remains constrained by the heavy organic framework and relatively low site density.

In parallel to carbon-based materials, boron-containing nanostructures have attracted significant attention due to their intrinsic electron deficiency and enhanced polarization effects, which can strengthen hydrogen binding. For example, Yuan et al. reported that doped porous boron nitride (2D BNC) can store up to 12 H_2_ molecules with a gravimetric capacity of 10.7 wt% [34]. Similarly, boron-doped carbon nanotubes and boron-substituted carbon frameworks have been investigated for hydrogen storage, as boron incorporation enhances charge redistribution and adsorption strength [35]. While these hybrid B–C systems show improved interaction compared to pristine carbon materials, their performance remains highly dependent on dopant concentration, structural defects, and synthetic feasibility, which complicates practical implementation.

Despite these advances, the identification of a lightweight, structurally stable, and electronically tunable quantum dot framework that simultaneously (i) prevents metal clustering, (ii) provides moderate Kubas-type adsorption, and (iii) supports high hydrogen loading remains an open challenge. In this context, boron–carbon quantum dots represent an unexplored class of materials combining low atomic mass, intrinsic polarity, and accessible coordination environments, potentially enabling enhanced metal stabilization and cooperative hydrogen adsorption.

Recently, Ahmed et al. introduced a novel C_3_B_2_ quantum dot (CBQD) with a trigonal bipyramidal geometry [36]. This zero-dimensional boron carbon cluster exhibits negative cohesive energy and real vibrational frequencies, confirming structural stability, and displays semiconducting behavior characterized by a finite HOMO–LUMO gap. However, to the best of our knowledge, no systematic investigation has yet examined hydrogen adsorption or transition-metal decoration of this boron–carbon quantum dot system.

In this context, the present work reports a comprehensive first-principles investigation of hydrogen storage on pristine and Ti-decorated C_3_B_2_ quantum dots. By combining DFT, high-level DLPNO-CCSD(T) benchmarking, energy decomposition analysis, and statistical thermodynamics modeling, we evaluate adsorption energetics, sequential hydrogen uptake, thermodynamic reversibility, and desorption kinetics under realistic operating conditions. This integrated theoretical approach provides new insights into the design of lightweight transition-metal-functionalized quantum dots for next-generation solid-state hydrogen storage applications.

### Computational Details

In the present study, the calculation strategy for the hydrogen storage on pristine and decorated C_3_B_2_ quantum dot was carried out in four steps. Firstly, we optimized the isolated systems structure. Then, we studied the adsorption mechanism of a single H_2_ molecule on the surface. After that, we studied the adsorption of sequential hydrogen molecules to determine the mass capacity of the quantum dot to store H_2_. Finally, we estimated the hydrogen uptake capacity at a suitable temperature and pressure. Geometries of both bare and hydrogen adsorbed C_3_B_2_ were optimized without any symmetry constraints using a higher-level correlated approaches such as DLPNO-CCSD(T) [37,38,39,40,41]. For the DFT calculations, a diverse selection of widely used and recently developed functionals was employed, encompassing generalized gradient approximation (GGA), hybrid GGA, and hybrid meta-GGA functionals, including B3LYP [42,43], PBE [44], CAM-B3LYP [45], M06-2X [46], ωB97X-V [47], and ωB97X-3c [48]. All computations utilized the correlation-consistent cc-pVTZ basis set of triple-ζ quality [49], except for those employing the ωB97X-3c functional, which relies on a polarized valence double-ζ (vDZP). The vDZP basis set minimizes and, in most cases, eliminates the basis set superposition error (BSSE), achieving accuracy comparable to that of triple-ζ basis sets, with residual errors effectively corrected by the D4 scheme [50]. Therefore, DNP is used to render the improved results for studying the weak non-covalent interactions and reaction coordinates, which play a crucial role in hydrogen sorption events. The selection of ωB97X-3c as the primary working functional was motivated by its composite design, which combines range separation, D4 dispersion correction, and geometrical counterpoise (gCP) correction to reduce basis set superposition error (BSSE). This functional has been shown to provide reliable geometries and interaction energies for non-covalent and transition-metal-containing systems at moderate computational cost. Benchmark comparison with DLPNO-CCSD(T)/cc-pVTZ confirms its accuracy for the present system. The use of cc-pVTZ basis sets further minimizes BSSE effects, and cross-validation across multiple functionals ensures robustness of the predicted adsorption trends. All calculations were carried out using the ORCA program [51,52,53,54], employing the DefGrid3 integration grid and TightOpt settings. For understanding the thermodynamic stability, the frequency was calculated using the same set of methods. Harmonic vibrational frequency calculations were carried out at the same level of theory as the geometry optimizations to verify the nature of the stationary points. All optimized structures were confirmed as true local minima, as no imaginary frequencies were observed. The calculated vibrational frequencies and corresponding infrared (IR) spectra are provided in the Supporting Information (See Tables S2 and S3).

The detailed characteristics of charge transfer were analyzed using the charge decomposition analysis (CDA) [55,56]. The calculations were carried out using the Multiwfn program [57,58].

The C_3_B_2_ system considered here represents a zero-dimensional quantum dot (finite cluster) rather than a bulk material. Consequently, surface and edge effects are intrinsic to the model and are expected to dominate the adsorption behavior. Although adsorption energies may vary with dot size, the robustness of the predicted trends is supported by cross-validation across multiple DFT functionals and benchmarking against DLPNO-CCSD(T) reference calculations (Table S1). Therefore, while finite-size effects may influence absolute values, the qualitative conclusions regarding reversibility and sequential hydrogen uptake remain reliable within the estimated methodological uncertainty.

## 2. Results and Discussion

### 2.1. Hydrogen Adsorption on Pristine C_3_B_2_ Quantum Dot

**Figure 1:**
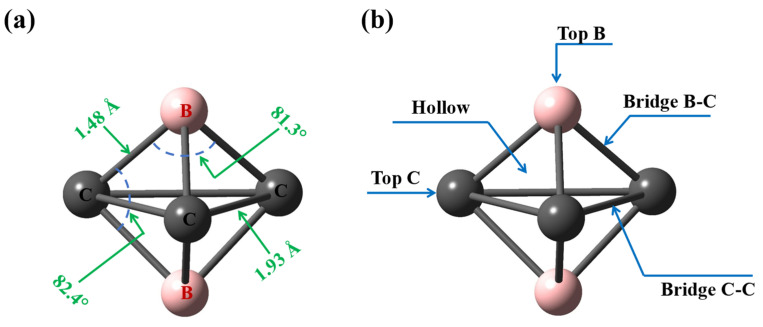
(**a**) Optimized atomic structure of C_3_B_2_ quantum dot using ωB97X-3c/vDZP level of theory and (**b**) their possible adsorption sites.

The starting geometry of the isolated C_3_B_2_ quantum dot (CBQD) obtained from previously reported literature source [36]. The CBQD is of trigonal bipyramidal molecular geometry, which describes a saddle-like shape formed by three carbon atoms and two boron atoms arranged in a shape resembling two triangular pyramids joined at their bases (Figure 1a).

The three C atoms form the triangular base and the two B atoms occupy the top of the pyramid. The analysis of the CBQD geometry using DLPNO-CCSD(T), along with various functionals, provides valuable insights into the accuracy and performance of different computational methods in describing the bond lengths and the bond angles (Table 1). The binding energy per atom $E_{b}$ is defined as:

(1) $$E_{b}=\frac{E_{\mathrm{CBQD}}-3E_{C}-2E_{B}}{5}$$

where $E_{\mathrm{CBQD}}$, $E_{C}$, and $E_{B}$ are the total energies of the C_3_B_2_ quantum dot, an isolated carbon atom, and an isolated boron atom, respectively.

The geometry of CBQD was successfully optimized using DLPNO-CCSD(T)/cc-pVTZ, ωB97M-V/QZVP, ωB97X-3c/vDZP, PBE/cc-pVTZ, M06-2X/cc-pVTZ, TPSSh/cc-pVTZ, and B3LYP-D4/cc-pVTZ. The corresponding binding energies (calculated from Equation (1)) are −3.85, −4.05, −3.85, −4.51, −4.15, −4.10, and −3.99 eV, respectively (Table 1). The negative binding energies confirm the intrinsic structural stability of CBQD. Among the evaluated functionals, ωB97X-3c shows the highest consistency with the DLPNO-CCSD(T) reference value, demonstrating its reliability for describing the electronic structure of this system. The ωB97X-3c method predicts B–B (1.958 Å), B–C (1.486 Å), and C–C (1.937 Å) bond lengths, along with B–C–B (82.8°) and C–B–C (81.3°) bond angles, within ~0.03 Å, 0.02 Å, 0.07 Å, 3.1°, and 2.4°, respectively, of the DLPNO-CCSD(T)/cc-pVTZ reference values. This excellent agreement demonstrates the high accuracy of ωB97X-3c in reproducing the optimized geometry of CBQD. The computed structural parameters are consistent with experimentally reported bond distances in boron–carbon frameworks and small boron-containing clusters. Covalent B–C single bonds in molecular and solid-state boron–carbon compounds typically fall within the range 1.48–1.55 Å, while B–B bonds in electron-deficient boron systems are commonly observed near 1.90–2.00 Å [59,60]. The calculated B–C distance of 1.486 Å and B–B distance of 1.958 Å therefore lie well within experimentally established values. The elongated C–C bond (1.937 Å) deviates from conventional sp^2^ C=C (1.34–1.40 Å) and sp^3^ C–C (1.53–1.55 Å) distances. However, such bond expansion is characteristic of highly strained polyhedral boron–carbon clusters and trigonal bipyramidal frameworks, where angular compression (~82°) induces significant bond stretching. Comparable structural distortions have been reported for small boron carbide clusters and boron-rich cage compounds [35]. These structural features indicate that the bonding pattern in CBQD is consistent with electron-deficient, partially multi-center interactions typical of boron-containing nanoclusters, rather than classical localized two-center covalent bonding. This behavior further supports the intrinsic stability of the trigonal bipyramidal C_3_B_2_ framework.

**Figure 2:**
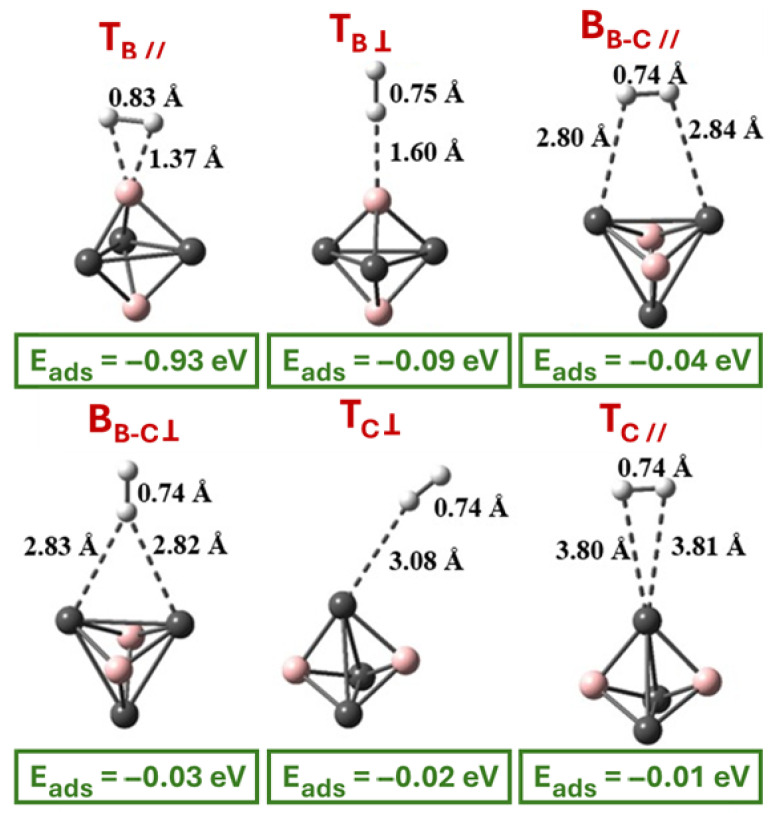
The most stable geometries of H_2_/CBQD complex optimized using wB97x-3c/vDZP level of theory. The green values represent the adsorption energies E_ads_ in eV and the notations in red are the names of the obtained configurations.

To examine the ability of CBQD to store molecular hydrogen, we introduced a single H_2_ molecule to the system’s surface. The adsorption simulation of the H_2_ molecule on CBQD was performed with the DFT-D4/wB97x-3c/vDZP approach in the gas phase. The H-H bond length of isolated H_2_ molecule is 0.742 Å, consistent with the experimental value of 0.74 Å and developed computational results [61,62]. According to the optimized geometry of the adsorbent, fully relaxed H_2_ molecule is directly added to each non-equivalent adsorption site of the CBQD with initial distance of 2.5 Å. Figure 1b shows the five possible adsorption sites on CBQD, namely Top B (top of the boron atom), Top C (top of the carbon atom), Hollow (triangle CBC center), Bridge B–C (bridge of B–C bond) and Bridge C–C (bridge of C–C bond). Accordingly, about twenty possible adsorption configurations of hydrogen molecule can be constructed, where for each site there are configurations with H_2_ parallel (∥) with and vertical (⊥) to the CBQD surface, respectively. Following the optimization of various possibilities for H_2_ adsorption on the CBQD surface, we identified six stable configurations, which are denoted T_B//_, T_B⊥_, B_B-C//_, B_B-C⊥_, T_C⊥_ and T_C//_ (Figure 2). To evaluate the interaction strength between the hydrogen molecule and the C_3_B_2_ quantum dot, the adsorption energy ($E_{\mathrm{ads}}$) was calculated using the following expression:

(2) $$E_{\mathrm{ads}}=E_{H_{2}/\mathrm{CBQD}}-[E_{\mathrm{CBQD}}+E_{H_{2}}]$$

where $E_{H_{2}/\mathrm{CBQD}}$ is the total electronic energy of the hydrogen-adsorbed C_3_B_2_ system, $E_{\mathrm{CBQD}}$ is the total energy of the isolated C_3_B_2_ quantum dot, and $E_{H_{2}}$ is the total energy of a free H_2_ molecule. A negative value of $E_{\mathrm{ads}}$ indicates an energetically favorable adsorption process, with larger magnitudes corresponding to stronger interactions between H_2_ and the substrate.

Six distinct configurations were identified, named according to the adsorption axis and the nature of the underlying atom (T_B//_, T_B⊥_, B_B-C//_, B_B-C⊥_, T_C⊥_ and T_C//_). The computed adsorption energies (E_ads_), shown in green in Figure 2, range from −0.93 eV (T_B//_) to −0.01 eV (T_C//_), revealing a strong site-dependence of the interaction strength. The most stable configuration is clearly T_B//_, in which H_2_ is aligned parallel to the B–C bond axis, indicating that boron atoms serve as the most reactive anchoring points for H_2_ on the CBQD surface.

A comparative benchmark study using five different DFT functionals (B3LYP-D4, TPSSH, M06-2X, wB97X-3c, wB97M-V) and high-level DLPNO-CCSD(T) calculations (Table S1) further validates this result. Across all methods, the adsorption energy of the most stable H_2_/CBQD complex lies in the narrow range −0.87 to −0.99 eV, confirming the robustness of the predicted interaction strength. The calculated B–H (1.33–1.37 Å), B–B (2.01–2.13 Å), and B–C (1.51–1.54 Å) distances are consistent across functionals and in excellent agreement with the CCSD(T) reference values. Importantly, the H–H bond stretches from 0.74 Å in free H_2_ to 0.84 Å upon adsorption, revealing a substantial weakening of the H–H bond that exceeds typical values for physisorption.

To gain deeper insight into the nature of this interaction, an energy decomposition analysis (EDA) was performed at the M06-2X level (Table 2). The attractive interaction energy totals −8.12 eV, dominated by polarization (−3.38 eV, 41.6%) and exchange (−2.59 eV, 31.9%), with smaller contributions from electrostatics (18.5%) and dispersion (8.0%). Such a large polarization term indicates strong deformation of the H_2_ electron cloud, accompanied by partial σ → σ* electron transfer. This electronic reorganization manifests structurally as H–H bond activation and the development of short B–H contacts (1.36–1.37 Å), which are longer than typical covalent B–H bonds (~1.19–1.21 Å) but consistent with weak or partially covalent B–H interactions reported for electron-deficient boron centers [59,63].

The repulsive Pauli term is notably large (+6.97 eV), suggesting substantial orbital overlap between H_2_ and the CBQD surface. The resulting total interaction energy of −1.15 eV unequivocally places the H_2_–CBQD interaction in the chemisorption regime, far beyond the optimal window for reversible hydrogen storage (−0.1 to −0.6 eV per H_2_) [7,64]. This indicates that pristine C_3_B_2_ strongly activates and partially dissociates H_2_, making desorption energetically demanding and therefore unsuitable for cyclic operation in practical storage systems.

**Figure 3:**
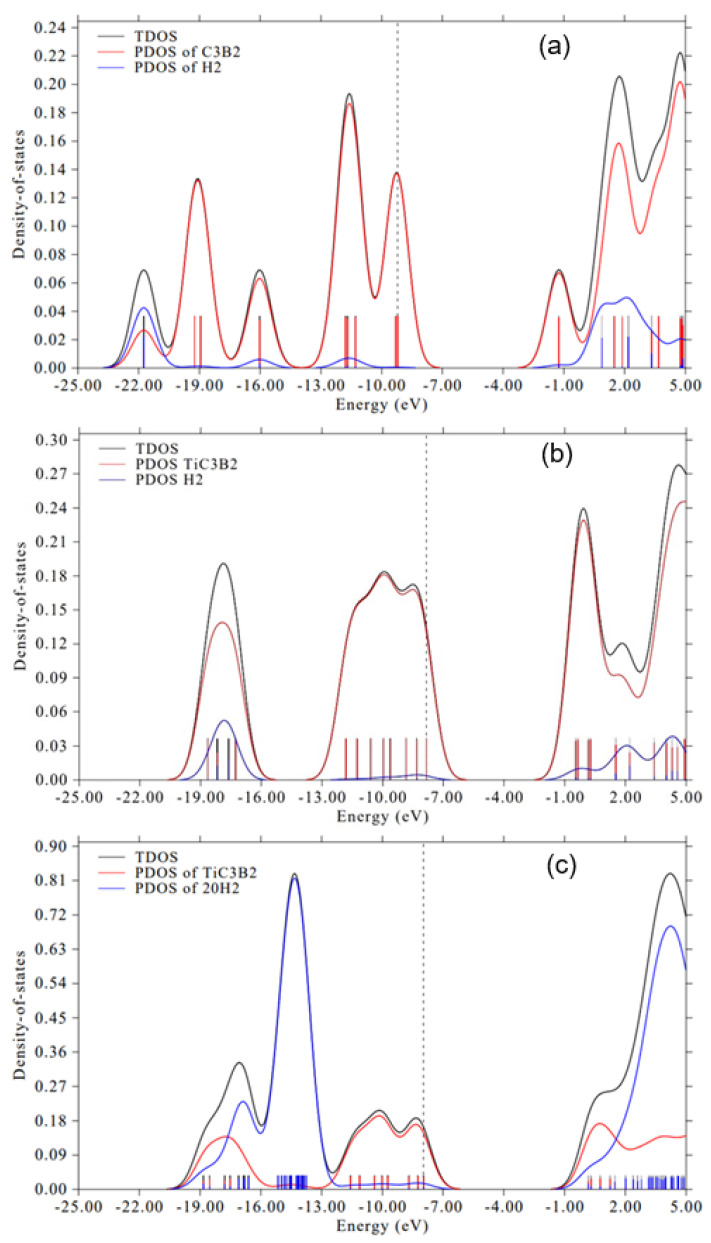
Total and projected density of states of (**a**) H_2_/CBQD, (**b**) H_2_/Ti−CBQD and (**c**) 20H_2_/Ti−CBQD systems.

To further clarify the nature of hydrogen interaction with pristine CBQD, total and projected density of states (TDOS/PDOS) analyses were performed (Figure 3a). The PDOS calculated over the energy range from −25 to 5 eV provides clear evidence of strong electronic interactions between the H_2_ molecule and the C_3_B_2_ cluster. Significant hybridization between hydrogen and CBQD orbitals is observed in the deep valence region, with pronounced overlapping centered at approximately −22 eV and additional hybridized states near −16 eV and −11.5 eV. These overlaps indicate the formation of stabilized bonding molecular orbitals arising from interaction between the H 1s orbitals and the valence states of the C and B atoms. Notably, the hydrogen states exhibit broadening and delocalization over a wide energy range, demonstrating the loss of molecular character and confirming strong orbital mixing with the C_3_B_2_ cluster. Furthermore, the presence of hydrogen contributions extending toward the Fermi level indicates significant redistribution of electron density and partial population of antibonding orbitals, consistent with activation and weakening of the H–H bond. These PDOS features are fully consistent with the EDA, which shows a large polarization contribution. The strong polarization term reflects significant distortion and reorganization of the electronic density upon adsorption. This correlation between PDOS and EDA confirms that hydrogen adsorption on pristine C_3_B_2_ is dominated by strong orbital interaction and polarization effects, leading to chemisorption with a large adsorption energy.

Overall, the combined geometric, energetic, and electronic analyses establish that pristine C_3_B_2_ binds H_2_ too strongly, through a polarization-driven chemisorption mechanism involving substantial H–H bond activation. These findings justify the need for metal decoration (e.g., Ti) to tune the electronic environment of the CBQD and achieve reversible molecular hydrogen adsorption, as discussed in the next section.

### 2.2. Hydrogen Storage on Ti Decorated C_3_B_2_ Quantum Dot

As reported in numerous studies, metal decoration is commonly employed to enhance the interaction energy between host materials and H_2_ molecules. However, our results reveal an opposite trend. The pristine C_3_B_2_ monolayer intrinsically exhibits a strong interaction with H_2_ (E_ads_ = −0.93 eV), which exceeds the optimal range for reversible hydrogen storage. Therefore, an effective approach to improve its storage performance is to reduce the adsorption energy to values within −0.10 eV to −0.60 eV, ensuring efficient adsorption–desorption cycling under ambient conditions. To achieve this, we explored the effect of metal decoration on the C_3_B_2_ surface using Ti atom. The choice of this metal is motivated by their distinct electronic properties and their proven ability to tune hydrogen adsorption in nanostructured materials. Titanium, on the other hand, is a transition metal capable of forming Kubas-type interactions with H_2_, resulting in an intermediate adsorption strength between physisorption and chemisorption. Ti decoration has been successfully applied to graphene, boron nitride nanosheets, and borophene, where Ti atoms induce d–orbital hybridization with H_2_, leading to reversible adsorption with favorable thermodynamic characteristics [65].

A comparative investigation of Ti-decorated C_3_B_2_ system thus provides valuable insights into how metal dopants can effectively modulate adsorption energetics, guiding the rational design of boron–carbon-based materials for efficient and reversible hydrogen storage applications.

**Figure 4:**
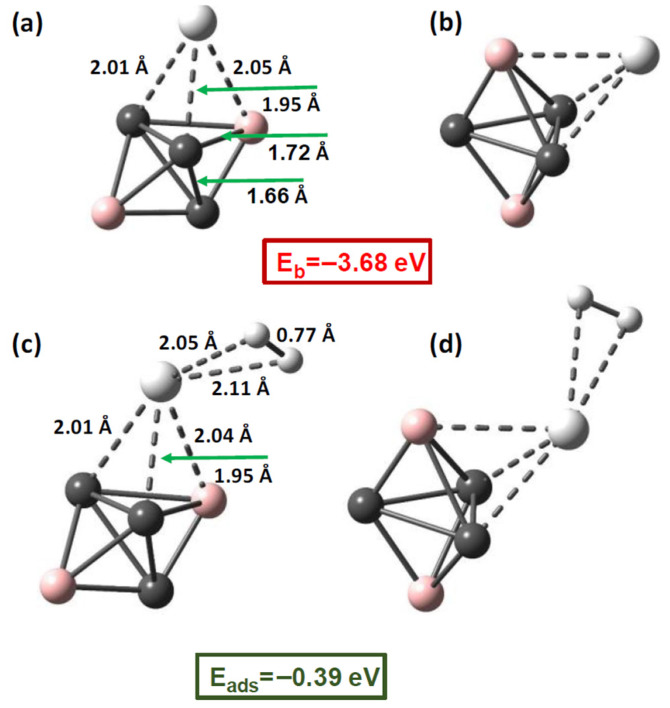
Optimized structures of Ti–C_3_B_2_ and Ti–C_3_B_2_–H_2_ at the ωB97X-3c/vDZP level. (**a**) Top view of Ti anchoring site, (**b**) Side view illustrating framework distortion, (**c**) Ti–H_2_ coordination geometry and (**d**) Alternative perspective highlighting Ti–H_2_ interaction distances.

The decoration of the C_3_B_2_ quantum dot (CBQD) was evaluated by examining all five plausible adsorption sites (Top B, Top C, Hollow, Bridge B–C, and Bridge C–C) using the DFT-D4/ωB97X-3c/vDZP approach. Among these configurations, the Hollow site, where the Ti atom is coordinated with two carbon atoms and one boron atom, was identified as the most stable (Figure 4). The optimized structure shows Ti–C bond lengths of 1.95 and 2.01 Å and a Ti–B bond length of 2.05 Å, corresponding to a strong adsorption energy of −3.68 eV. Upon Ti anchoring, noticeable structural modifications occur within the CBQD framework, including contraction of the C–C bond to 1.66 Å and elongation of the B–C bond to 1.72 Å. The calculated Ti–C and Ti–B distances are consistent with experimentally characterized Ti–carbon and Ti–boron coordination environments. In molecular Ti–alkyl and Ti–aryl complexes, Ti–C bond lengths typically range between 1.95 and 2.10 Å depending on oxidation state and coordination number [59]. Similarly, Ti–B interactions in organoborane and metal–boron cluster systems are commonly observed in the 2.00–2.20 Å range, reflecting polarized covalent bonding. The coordination geometry around Ti in Ti–CBQD resembles a distorted three-fold environment imposed by the trigonal bipyramidal host framework. The observed bond distortions further indicate substantial electronic reorganization upon Ti anchoring, consistent with strong metal–substrate hybridization. Comparable structural rearrangements have been reported for Ti-decorated carbon nanostructures and boron-containing clusters [28]. These structural parameters support a partially covalent Ti–substrate interaction rather than weak physisorption, explaining the high anchoring energy (−3.68 eV) and the strong resistance to Ti migration or clustering. Such structural and energetic features support a partially covalent Ti–substrate interaction rather than weak physisorption, explaining both the large anchoring energy (−3.68 eV) and the enhanced resistance to Ti migration or clustering. For comparison, reported Ti adsorption energies on graphene and borophene typically range from −2.5 to −3.5 eV [66,67]. The slightly stronger binding observed for Ti–CBQD places it at the upper end of this range and is consistent with increased local polarization and curvature effects inherent to the quantum dot framework.

These results indicate that the Ti atom binds strongly to the CBQD surface, exhibiting an adsorption energy that exceeds those reported for extended two-dimensional materials. The enhanced binding energies on CBQD can be attributed to the increased chemical reactivity of the quantum dot system, which provides under coordinated sites favoring metal anchoring. Such strong metal substrate interactions are advantageous for stabilizing dispersed metal atoms and preventing clustering, a critical aspect for practical applications in hydrogen storage.

In contrast to extended boron-doped graphene and borophene sheets, the discrete zero-dimensional structure of C_3_B_2_ provides localized coordination environments and enhanced curvature-induced polarization effects. These features facilitate stronger Ti anchoring and reduce the likelihood of metal aggregation, thereby offering improved stability of isolated metal centers compared to conventional two-dimensional substrates. In addition to suppressing Ti aggregation, maintaining low Ti coverage is also important for preserving the structural integrity and adsorption regime of CBQD. Our tests with two Ti atoms on this ultrasmall quantum dot lead to pronounced framework distortion, indicating that higher coverage is not structurally favorable. The dimerized configuration does not provide additional energetic stabilization compared to the isolated single-Ti decoration and induces significant geometric strain, indicating that single-atom decoration represents the thermodynamically and structurally preferred state for this ultrasmall quantum dot. Moreover, single-Ti decoration already achieves the key design objective of this work: it tunes the interaction from overly strong adsorption on pristine CBQD (chemisorption-like binding) to a moderate, reversible molecular adsorption regime. Therefore, we focus on the single-Ti configuration as the most chemically meaningful and practically relevant decoration state for reversible hydrogen storage on CBQD.

The adsorption of H_2_ on the Ti-decorated C_3_B_2_ quantum dot (Ti–CBQD) was investigated to elucidate the influence of metal functionalization on the interaction mechanism and binding strength. To ensure identification of the global minimum, we systematically tested all plausible adsorption sites, including the Ti center as well as B and C framework sites, considering multiple initial orientations of the H_2_ molecule. All optimized geometries obtained at the DFT-D4/ωB97X-3c/vDZP level consistently converged toward a unique minimum-energy configuration centered at the Ti atom, characterized by an adsorption energy of −0.39 eV (Figure 4). In contrast, adsorption on the carbon site was found to be very weak (−0.01 eV), while initial placement near boron relaxed to a bridge-like Ti–H_2_–B configuration with a significantly smaller stabilization (−0.11 eV), indicating that the Ti center dominates the interaction. These results confirm that H_2_ preferentially binds to the Ti site in the decorated system. The adsorption energy of −0.39 eV lies within the thermodynamic window typically associated with reversible hydrogen storage (−0.10 to −0.60 eV), indicating that Ti decoration effectively moderates the excessive chemisorption observed on the pristine C_3_B_2_ surface.

The optimized structure shows that the H_2_ molecule remains molecular upon adsorption, with its bond length increasing only slightly from 0.74 Å in the gas phase to 0.77 Å. This moderate elongation indicates weakening of the H–H σ bond without dissociation. The Ti–H_2_ distances (2.05–2.11 Å) are consistent with η^2^-H_2_ coordination geometries reported for early transition-metal dihydrogen complexes. In literature-reported Ti dihydrogen complexes, Ti–H_2_ distances typically range from 1.9 to 2.2 Å, while H–H bond lengths increase from 0.74 Å in free H_2_ to approximately 0.78–0.82 Å depending on the extent of metal-to-σ* back-donation [61,68]. We also emphasize that a quantitative comparison with representative Ti-decorated 2D materials and nanocage hosts is provided in Table 3.

The structural parameters obtained here therefore fall within the established regime of non-dissociative dihydrogen coordination. These features confirm that hydrogen remains molecularly bound to the Ti center through a balanced σ-donation from H_2_ to the metal and π-backdonation into the H_2_ σ* orbital, characteristic of a Kubas-type interaction. Such a mechanism explains the moderate adsorption energy and supports the reversibility of the hydrogen storage process.

This interpretation is strongly supported by the energy decomposition analysis (EDA) performed at the M06-2X level. The total attractive contribution amounts to −2.03 eV, with electrostatics, exchange, polarization, and dispersion contributing in a comparable manner. The near-uniform distribution of these attractive terms shows that no single interaction dominates the binding; instead, the stabilization results from a cooperative interplay of moderate contributions. The repulsion term, quantified at 1.63 eV, counterbalances the attractive components, yielding a final interaction energy of −0.40 eV, in excellent agreement with the DFT-D4 result.

The balanced character of the EDA terms and the moderate H–H bond elongation unambiguously point to a Kubas-type adsorption mechanism. In this regime, electron density is partially transferred from the σ orbital of H_2_ to the vacant Ti d-orbitals (σ-donation), while simultaneous backdonation from filled Ti d-states into the σ* antibonding orbital of H_2_ weakens the bond without inducing dissociation. Such a dual orbital interaction explains both the stability of the H_2_ molecule and the observed elongation of its bond. These results confirm that the Ti center operates as an electron mediator rather than as a dissociative catalyst, which is a critical requirement for reversible hydrogen sorption.

To further elucidate the Ti-mediated interaction, the TDOS/PDOS of H_2_/Ti–CBQD is presented in Figure 3b. A clear overlap between H 1s and Ti 3d states is observed in the −19 to −16 eV region and near the Fermi level. This hybridization reflects σ-donation from the H_2_ bonding orbital to empty Ti d states, accompanied by partial back-donation into the H_2_ σ* orbital. Unlike pristine C_3_B_2_, hydrogen states remain relatively localized, indicating preservation of molecular character. These features provide direct electronic evidence of a Kubas-type interaction.

A comparative analysis of Ti–CBQD with representative Ti-decorated materials presented in Table 3 further highlights the relevance of the present system within the broader family of lightweight hydrogen hosts. The adsorption energy obtained for Ti–C_3_B_2_ (−0.39 eV) lies squarely within the range reported for other Ti-functionalized 2D materials such as graphene (−0.35 to −0.55 eV), borophene (−0.35 to −0.60 eV), and h-BN (−0.30 to −0.50 eV). These systems similarly preserve the molecular nature of H_2_, displaying moderate H–H bond elongations of 0.77–0.80 Å and characteristic Ti–H distances close to ~2.0–2.2 Å, consistent with non-dissociative dihydrogen binding modes.

Comparable trends are also observed in zero-dimensional nanocage hosts such as Ti–C_24_ and Ti–B_12_N_12_, which show slightly stronger adsorption energies (−0.30 to −0.70 eV) and H–H distances in the 0.78–0.83 Å range, reflecting the enhanced curvature and local polarization inherent to cage-type structures. These nanocage systems are widely recognized for supporting molecular H_2_ adsorption at moderate binding strengths, and the agreement with Ti–CBQD indicates that the C_3_B_2_ quantum dot offers a similarly favorable electronic environment.

Finally, the behavior of Ti–CBQD also aligns with that reported for Ti–MOF-74, a well-studied porous framework displaying adsorption energies between −0.15 and −0.25 eV and short H–H distances around ~0.75 Å. Although weaker, these values still correspond to reversible molecular adsorption dominated by electrostatic and weak Kubas interactions.

Overall, the comparison demonstrates that the adsorption properties of Ti–C_3_B_2_ are consistent with those of established Ti-decorated 2D substrates, nanocages, and porous frameworks. This confirms that the C_3_B_2_ quantum dot provides an appropriate balance of curvature, polarization, and metal coordination to stabilize H_2_ within the optimal regime for reversible storage.

### 2.3. Sequential Hydrogen Adsorption and Reversible H_2_ Storage on Decorated CBQD

To evaluate the overall hydrogen storage performance of Ti-decorated C_3_B_2_ (Ti-CBQD), successive H_2_ molecules were incrementally introduced onto the most stable adsorption configuration. All structures were optimized at the DFT-D4/ωB97X-3c/vDZP level of theory, and the corresponding adsorption energies, bond distances, and gravimetric capacities were analyzed to provide insight into the adsorption mechanism, thermodynamic reversibility, and kinetic feasibility of the system. The average adsorption energy per hydrogen- molecule was obtained from:

(3) $${\bar{E}}_{\mathrm{ads}}=\frac{E(nH_{2}/\mathrm{CBQD}\text{–}\mathrm{Ti})-[E(\mathrm{CBQD}\text{–}\mathrm{Ti})+nE(H_{2})]}{n}$$

where $E(nH_{2}/Ti-\mathrm{CBQD})$ is the total electronic energy of the Ti-decorated system containing *n* hydrogen molecules, E(Ti−CBQD) is the total energy of the isolated substrate, and $E(H_{2})$ is the energy of an isolated H_2_ molecule.

The computed average adsorption energies per H_2_ molecule and structural parameters are summarized in Table 4. The average adsorption energy per H_2_ molecule decreases smoothly from −0.39 eV/H_2_ for the first molecule to −0.11 eV/H_2_ for the twentieth, showing a gradual filling of the Ti coordination environment and mild H_2_–H_2_ repulsion as the surface becomes saturated. All values remain within the DOE-recommended window (−0.10 to −0.60 eV/H_2_), confirming that hydrogen adsorption on Ti–CBQD is strong enough for stable retention but weak enough for easy release under near-ambient conditions.

Importantly, structural analysis reveals two distinct adsorption regimes. As shown in the optimized geometries provided in Figure S1 (Supporting Information), the Ti center becomes saturated at approximately five H_2_ molecules. These first five hydrogen molecules are directly coordinated to the Ti atom through non-dissociative Kubas-type interactions, characterized by Ti–H_2_ distances of ~2.05–2.15 Å and moderate H–H elongation (~0.77 Å). This coordination number is fully consistent with the established maximum H_2_ coordination capacity of Ti complexes (typically ≤6).

Beyond five H_2_ molecules, additional hydrogen does not directly coordinate to the Ti center. Instead, the extra H_2_ molecules occupy peripheral regions around the Ti–C_3_B_2_ quantum dot and are stabilized through cooperative interactions involving surface polarization, dispersion forces, and H_2_–H_2_ packing effects. The complete structural evolution from *n* = 1 to *n* = 20 is provided in Figure S1 (Supporting Information), which clearly distinguishes Ti-site saturation from overall cluster loading.

The gravimetric hydrogen storage capacity (wt%) was determined using:

(4) $$\mathrm{wt}\%=\frac{nM(H_{2})}{M(Ti-\mathrm{CBQD})+nM(H_{2})}\times 100$$

where $M(H_{2})$ and M(Ti−CBQD) are the molar masses of hydrogen and Ti-decorated C_3_B_2_, respectively.

For five directly coordinated H_2_ molecules (Ti-site saturation), the corresponding gravimetric capacity is 8.72 wt%, which already exceeds the DOE target of 5.5 wt%. Upon further loading of peripheral hydrogen molecules, the total hydrogen content increases nearly linearly, reaching a maximum theoretical system capacity of 27.49 wt% at *n* = 20.

It is essential to emphasize that this maximum value refers to the total hydrogen loading of the Ti–C_3_B_2_ unit as a whole and does not imply that all 20 H_2_ molecules are directly coordinated to the Ti center. Rather, it reflects the combined contribution of Ti-site Kubas adsorption and cooperative cluster adsorption enabled by the lightweight boron–carbon framework.

The corresponding H–H bond lengths (0.75–0.77 Å) confirm that hydrogen remains molecularly adsorbed at all coverages, with no evidence of dissociation (Table 4). This preservation of molecular character indicates that adsorption proceeds through non-dissociative metal–dihydrogen coordination combined with weaker surface-mediated interactions at higher loading. The consistent Ti–H_2_ distances and absence of significant geometric distortion further demonstrate that the Ti active site remains structurally and electronically stable even at full hydrogen coverage.

Overall, the Ti–C_3_B_2_ quantum dot exhibits a dual-mode hydrogen storage mechanism: (i) primary Ti-centered Kubas coordination up to approximately five H_2_ molecules and (ii) secondary cooperative adsorption over the quantum dot surface. The persistence of average adsorption energies within the reversible thermodynamic window across the entire loading range confirms that hydrogen uptake remains energetically favorable while ensuring practical desorption.

For the fully loaded 20H_2_/Ti–C_3_B_2_ system (Figure 3c), the PDOS analysis reveals a substantial increase in hydrogen-derived states across the valence region, reflecting the high storage capacity. However, the degree of hybridization between H 1s and Ti 3d orbitals per hydrogen molecule decreases compared to the single-H_2_ configuration, consistent with partial saturation of available Ti d states. Importantly, no significant delocalization of hydrogen states toward dissociative antibonding features is observed, confirming that hydrogen retains its molecular integrity even at maximum loading. These electronic signatures provide further support for the cooperative adsorption mechanism deduced from structural analysis.

Because efficient hydrogen storage materials must not only adsorb hydrogen effectively but also release it under mild conditions, the desorption energy ($E_{\mathrm{des}}$) was calculated to quantify the energy barrier associated with hydrogen release and to assess the reversibility of the storage process. To ensure thermodynamic reversibility, the desorption energy was calculated as follows [69]:

(5) $$E_{\mathrm{des}}=E_{nH_{2}/\mathrm{CBQD}\text{–}\mathrm{Ti}}-[E_{(n-1)H_{2}/\mathrm{CBQD}\text{–}\mathrm{Ti}}+E_{H_{2}}]$$

In this equation, $E_{nH_{2}/\mathrm{CBQD}\text{–}\mathrm{Ti}}$ is the total electronic energy of the Ti–C_3_B_2_ system containing *n* hydrogen molecules, $E_{(n-1)H_{2}/Ti-\mathrm{CBQD}\text{–}\mathrm{Ti}}$ corresponds to the energy of the same system after one H_2_ molecule has been desorbed, and $E_{H_{2}}$ is the energy of an isolated hydrogen molecule in the gas phase. A smaller $E_{\mathrm{des}}$ value implies that hydrogen can be released more easily, indicating a reversible storage process suitable for cyclic operation.

As illustrated in Table 4, both adsorption and desorption energies exhibit a smooth, nearly linear evolution with increasing number of adsorbed H_2_ molecules on the Ti–C_3_B_2_ quantum dot. The average adsorption energy ${\bar{E}}_{\mathrm{ads}}$ gradually increases from −0.39 eV to −0.11 eV per H_2_ as coverage increases, indicating a progressive weakening of interaction due to site saturation and intermolecular repulsion among adjacent H_2_ molecules. In contrast, the desorption energies ($E_{des}$) remain slightly less negative (by ~0.05 eV), confirming that hydrogen release requires only minimal thermal activation. This narrow energy gap between adsorption and desorption processes demonstrates the thermodynamic reversibility and optimal binding strength of the Ti–C_3_B_2_ system, making it an excellent candidate for practical hydrogen storage applications under ambient conditions. Such a feature is highly desirable because it minimizes energy loss between storage and release cycles and ensures long-term system stability. However, the Ti–C_3_B_2_ offers a higher hydrogen loading capacity, confirming that the boron–carbon quantum dot framework efficiently stabilizes Ti atoms and facilitates reversible molecular adsorption without significant structural deformation.

Beyond adsorption and desorption energetics, the thermal behavior and kinetic response of hydrogen release are crucial parameters that determine the practical feasibility of any solid-state storage system. Even when the adsorption energy lies within the ideal thermodynamic window, excessively high desorption temperatures or slow desorption rates can severely limit real-world applicability. Furthermore, to assess the practical feasibility of hydrogen uptake and release under real working environments, the adsorption–desorption behavior was extended to operational temperature and pressure conditions. The thermodynamic parameters were analyzed using statistical models based on the van’t Hoff and Arrhenius relations, enabling estimation of the effective desorption temperature and time for the H_2_/Ti–C_3_B_2_ system. This step bridges the atomistic-level adsorption energetics with the macroscopic performance required for real-world hydrogen storage applications.

Therefore, evaluating both the desorption temperature ($T_{D}$) and the desorption time (τ) provides valuable insights into the operational conditions required for hydrogen release and into the reversibility and cycling stability of the Ti–CBQD system.

To assess the desorption thermodynamics, the desorption temperature ($T_{D}$) was estimated using the van’t Hoff equation [70,71]:

(6) $$T_{D}=-\frac{{\bar{E}}_{\mathrm{ads}}}{k_{B}\;(\frac{\Delta S}{R}-lnP)}$$

T_D_ incorporates variables such as the average adsorption energy per H_2_ molecule (${\bar{E}}_{\mathrm{ads}}$), the equilibrium pressure (P), and ΔS, which represents the entropy change associated with the transition of hydrogen from the gas phase to the adsorbed state. Computational and experimental studies of H_2_ adsorption on carbon-based materials report adsorption entropy changes in the range of −75 to −80 J mol^−1^ K^−1^ at ~298 K, reflecting the loss of translational and rotational degrees of freedom upon adsorption while retaining vibrational contributions [72]. Accordingly, a magnitude of ΔS = 75.44 J mol^−1^ K^−1^ was adopted in the van’t Hoff analysis. This value is consistent with established statistical thermodynamics treatments of molecular adsorption processes [73,74]. Additionally, k_B_ and R represent the Boltzmann constant and the ideal gas constant, respectively. The maximum van’t Hoff temperature for desorption and the minimum van’t Hoff temperature for desorption are denoted as T_Dmax_ and T_Dmin_, respectively. These values are calculated by incorporating the highest and lowest of adsorption energy, respectively [69]. To specify the optimum temperature for desorption, we have quantified the average van’t Hoff temperature for desorption (T_D_) by taking an average of T_Dmax_ and T_Dmin_. The values for maximum, minimum and the average desorption temperatures with respect to a given pressure range are given in Table 5.

These results show that the desorption temperature increases with pressure, as expected from van’t Hoff thermodynamics. At low to moderate pressures (1–3 atm), the average desorption temperature is in the range 322–366 K (≈50–90 °C), which is compatible with practical, low-energy hydrogen release. At higher pressures (30–100 atm), higher temperatures are required (≈500–650 K), indicating that the system remains stable under storage conditions but can still be regenerated thermally.

Next, to evaluate the desorption kinetics, the desorption time (*τ*) is another critical parameter in evaluating the performance of hydrogen storage materials, as it reflects how rapidly hydrogen molecules can be released from the adsorbent surface. The desorption time can be estimated using the following equation [75]:

(7) $$\tau =\frac{\exp\left(-\frac{{\bar{E}}_{\mathrm{ads}}}{k_{B}T}\right)}{\nu_{0}}$$

The pre-exponential factor ν_0_ was taken as 10^12^ s^−1^, corresponding to a typical vibrational frequency of surface-adsorbed species within the framework of transition-state theory. For molecular hydrogen adsorbed on carbon-based and metal-decorated surfaces, reported attempt frequencies generally fall in the range of 10^11^–10^13^ s^−1^, reflecting characteristic H–surface stretching modes [72,75,76].

Since τ depends mainly on temperature (and only weakly on pressure in this model), it is more consistent to present it as a function of temperature, as shown in Table 5.

Table 5 clearly shows the expected Arrhenius-type behavior: the higher the temperature, the shorter the desorption time [77]. At 233 K, desorption is relatively slow (millisecond scale), which corresponds to a storage regime. Around room temperature (298 K), desorption already becomes fast (microsecond scale), and at slightly elevated temperatures (358–400 K), hydrogen release occurs in the sub-microsecond to nanosecond range. Such ultrafast kinetics mean that, once the activation temperature is reached, the Ti–CBQD system can deliver hydrogen almost instantaneously, a very desirable feature for on-demand hydrogen supply.

To summarize, Ti–CBQD can release hydrogen at moderate temperatures under realistic pressures, while the release is not only thermodynamically feasible but also kinetically very rapid. Together, these results reinforce the conclusion that hydrogen adsorption on Ti–C_3_B_2_ is fully reversible and suitable for repeated storage release cycles.

After confirming the favorable adsorption and desorption characteristics of Ti–CBQD, it is essential to evaluate its hydrogen storage performance under practical operational conditions, where both temperature and pressure influence the equilibrium between the adsorbed and gas phases. This step provides a direct connection between the microscopic DFT-derived adsorption parameters and the macroscopic hydrogen uptake expected under realistic working environments. Following the DFT calculations, hydrogen storage at operational conditions was evaluated using statistical thermodynamics within the grand canonical ensemble. This approach provides a quantitative link between the microscopic adsorption energies and the macroscopic hydrogen uptake under realistic temperature and pressure.

The grand canonical partition function (*z*) is defined as follows [78]:

(8) $$z=1+\sum_{i=1}^{n}exp(\frac{{\bar{E}}_{i,\mathrm{ads}}-\mu (P,T)}{k_{B}T})$$

where *n* is the maximum number of adsorbed H_2_ molecules, ${\bar{E}}_{i,\mathrm{ads}}$ is the average adsorption energy per H_2_ molecule of the *i*th H_2_ molecule, $k_{B}$ is the Boltzmann constant (1.38 × 10^−23^ J K^−1^), and μ(P,T) is the chemical potential of molecular hydrogen in the gas phase at a given pressure (*P*) and temperature (*T*).

The chemical potential μ(P,T) can be determined from thermodynamic relations or from experimental data, and it is given by:

(9) $$\mu_{H_{2}}(P,T)=\Delta H+T\Delta S+k_{B}Tln\frac{P}{P_{0}}$$

where ΔH, ΔS, and $P_{0}$ represent the enthalpy change, entropy change, and standard atmospheric pressure (1.01 × 10^5^ Pa), respectively. In this expression, the term ΔH+TΔS describes the free energy contribution of the hydrogen gas phase, while the logarithmic term accounts for the dependence on external pressure. The values of ΔH and ΔS were taken from the experimental database [79], which provides reliable thermodynamic parameters for H_2_ adsorption and desorption on metal-decorated systems.

**Figure 5:**
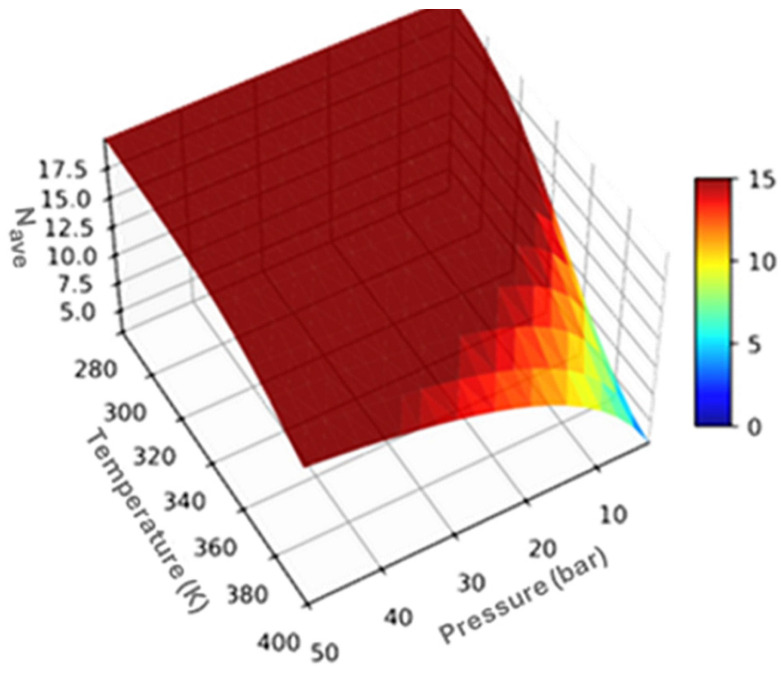
Three-dimensional surface plot of the average hydrogen loading (N_av_g) on Ti–C_3_B_2_ quantum dots as a function of temperature and pressure obtained from grand canonical ensemble calculations.

The average number of adsorbed hydrogen molecules at a given pressure and temperature, $N_{\mathrm{avg}}(P,T)$, is derived from the partition function and is expressed as:

(10) $$N_{\mathrm{avg}}(P,T)=N_{0}[\frac{z-1}{z}]$$

where $N_{0}$ denotes the number of adsorbed H_2_ molecules at 0 K (corresponding to the maximum adsorption capacity determined from DFT (20 H_2_ molecules). Figure 5 represents the average number of H_2_ molecules (N_avg_) adsorbed at the given P and T (i.e., N-P-T diagram).

Using this model, the operational hydrogen uptake was computed at practical adsorption and desorption conditions specifically, 30 atm and 298 K for adsorption, and 3 atm and 373 K for desorption. The results reveal that under these conditions, the number of adsorbed and desorbed hydrogen molecules are $N_{a}=19.89\;molecules$ and $N_{d}=6.62\;molecules$, respectively, leading to a reversibly stored hydrogen quantity $N_{p}=13.27\;molecules$. The corresponding effective gravimetric storage capacity is therefore C_E_ = 20.10 wt%.

The results obtained under operational conditions highlight the remarkable hydrogen storage performance of Ti–CBQD, achieving an effective reversible capacity of 20.10 wt%, which is considerably higher than most reported solid-state systems. This outstanding performance results from the synergy between moderate adsorption energies (−0.10 to −0.60 eV/H_2_) and ultrafast desorption kinetics, ensuring efficient adsorption–desorption cycling without structural degradation or energy loss.

A broader comparison with representative state-of-the-art hydrogen storage materials is presented in Table 6.

A broader comparison with representative nanostructured and solid-state hydrogen storage materials is summarized in Table 6. As shown, most Ti-decorated two-dimensional materials such as graphene, carbon nanotubes, and h-BN generally exhibit practical hydrogen storage capacities in the range of 4–8 wt%, while defect-engineered carbon systems and porous frameworks such as defected graphene, R-graphyne-MOF, and MOF-519 can reach approximately 8–12 wt% under favorable conditions. Similarly, covalent organic frameworks and related porous materials typically remain below 10 wt%, despite the presence of metal-functionalized adsorption sites. Among lightweight nanostructures, only a limited number of systems have been reported to exceed 20 wt%. For example, Sc-decorated irida-graphene reaches 21.6 wt%, while Mg-decorated C_8_ quantum dots were previously predicted to store 21.7 wt%. However, these systems rely on either less common substrate architectures or different adsorption environments. In contrast, Ti-decorated C_8_ quantum dots exhibit only 3.1 wt%, indicating that Ti alone does not guarantee high storage performance and that the host framework plays a decisive role.

The present Ti–C_3_B_2_ quantum dot achieves an effective reversible hydrogen storage capacity of 20.10 wt%, placing it among the highest-performing lightweight nanostructured systems reported to date. This remarkable performance results from the combined effect of strong Ti anchoring, the intrinsic polarization of the boron–carbon framework, and the dual adsorption mechanism involving primary Ti-centered Kubas coordination followed by cooperative surface adsorption.

Compared with conventional hydrides and porous frameworks, Ti–C_3_B_2_ simultaneously satisfies the three key requirements for efficient hydrogen storage: high gravimetric density exceeding the U.S. DOE target, moderate adsorption strength within the reversible thermodynamic window, and favorable desorption kinetics under near-ambient operating conditions. These combined features highlight Ti–C_3_B_2_ quantum dots as a highly competitive platform for next-generation reversible hydrogen storage.

## 3. Conclusions

This study establishes Ti-decorated C_3_B_2_ quantum dots as a highly effective, design-tunable platform for next-generation reversible hydrogen storage. High-level DFT and DLPNO-CCSD(T) calculations demonstrate that pristine C_3_B_2_ binds hydrogen too strongly, but Ti functionalization precisely modulates the adsorption energy into the optimal window for reversible sorption. The resulting system enables stable molecular adsorption with partially Kubas-type character, where the Ti center directly coordinates up to five H_2_ molecules, while additional hydrogen molecules are stabilized over the quantum dot surface through cooperative interactions. The system supports up to 20 H_2_ molecules per Ti–C_3_B_2_ unit, achieving an exceptional gravimetric capacity of 27.49 wt%. Thermodynamic and kinetic assessments reveal moderate desorption temperatures and ultrafast H_2_ release rates, confirming that the material can operate efficiently under realistic storage conditions. The effective reversible capacity of 20.10 wt%, obtained at 30/3 atm and near-ambient temperatures, significantly exceeds current DOE targets and surpasses many state-of-the-art nanostructured hydrogen storage media.

Beyond delivering high capacity, the C_3_B_2_ framework offers intrinsic advantages for materials engineering: low atomic mass, strong Ti anchoring preventing clustering, accessible adsorption sites, and structural robustness. These attributes underscore the potential of Ti–C_3_B_2_ quantum dots as a practical, lightweight, and scalable solid-state hydrogen storage material.

Overall, the present work provides clear design guidelines for tailoring boron–carbon quantum dots through transition-metal functionalization and highlights a promising direction for the development of high-performance hydrogen storage technologies. Although experimental synthesis of Ti-decorated C_3_B_2_ quantum dots has not yet been reported, the strong Ti anchoring energy and structural stability predicted here suggest that single-atom deposition strategies such as atomic layer deposition or wet-chemical methods could enable experimental realization. The present results therefore provide theoretical guidance for future synthesis and validation efforts.

Future work will focus on extending the present model to larger C_3_B_2_-based assemblies and evaluating the influence of multi-metal loading and Ti–Ti interactions on hydrogen storage performance. In addition, finite-temperature ab initio molecular dynamics simulations will be conducted to further assess the dynamic stability and practical feasibility of Ti-decorated boron–carbon quantum dots under realistic operating conditions.

## Figures and Tables

**Table 1:** Calculated bond lengths, bond angles, and binding energy of CBQD using selected DFT functionals and DLPNO-CCSD(T).

Methods	B–B (Å)	B–C (Å)	C–C (Å)	B–C–B (°)	C–B–C (°)	E_b_ (eV)
**B3LYP-D4**	1.898	1.496	2.003	78.7	84.1	−3.99
**TPSSh**	1.911	1.497	1.996	79.3	83.6	−4.10
**M06-2X**	1.993	1.483	1.902	84.4	79.8	−4.15
**PBE**	1.922	1.504	2.005	79.4	83.6	−4.51
**ωB97X-3c**	1.958	1.486	1.937	82.4	81.3	−3.85
**ωB97M-V-QZVP**	1.966	1.486	1.931	82.8	81.1	−4.05
**DLPNO-CCSD(T)**	1.928	1.511	2.016	79.3	83.7	−3.85

**Table 2:** Energy decomposition analysis (EDA) values for the H_2_/CBQD and H_2_/Ti–CBQD systems, calculated at the DFT/M06-2X level of theory.

Energy Terms (eV)	H_2_/CBQD	H_2_/Ti–CBQD
Electrostatic	−1.50	−0.44
Exchange	−2.59	−0.51
Polarization	−3.38	−0.58
Dispersion	−0.65	−0.42
Total attractive energy terms	−8.12	−2.03
Repulsion	6.97	1.63
Total interaction energy	−1.15	−0.40

**Table 3:** Comparison of DFT-predicted hydrogen storage capacities and adsorption energies of Ti-decorated nanostructures reported in the literature.

System	E_ads_ (eV)	d_(H–H)_ (Å)	d_(Ti–H2)_ (Å)	Reference
Ti-decorated C_3_B_2_	−0.39	0.77	2.05–2.11	This work
Ti-decorated graphene	−0.35 to −0.55	0.77–0.80	~2.0	[28]
Ti-decorated borophene	−0.35 to −0.60	0.77–0.80	2.0–2.1	[29]
Ti-decorated h-BN	−0.30 to −0.50	0.77–0.79	2.0–2.2	[30]
Ti–C_24_ nanocage	−0.30 to −0.60	0.78–0.82	2.05–2.15	[31]
Ti–B_12_N_12_ nanocage	−0.35 to −0.70	0.79–0.83	2.0–2.2	[32]
Ti–MOF-74	−0.15 to −0.25	~0.75	2.2–2.4	[33]

**Table 4:** Calculated average adsorption energy per H_2_ molecule ${\bar{E}}_{\mathrm{ads}}$ in eV/H_2_, desorption energy E_des_ in eV, the bond length after adsorption d_(H–H)_ in Å and theoretic gravimetric capacity as a function of adsorbed hydrogen number *n*, obtained at the D4/wB97x-3c/vDZP level of theory.

*n* (H_2_)	${\bar{E}}_{\mathrm{ads}}$ (eV/H_2_)	E_des_ (eV)	H–H (Å)	wt%	*n* (H_2_)	${\bar{E}}_{\mathrm{ads}}$ (eV/H_2_)	E_des_ (eV)	H–H (Å)	wt%
1	−0.39	-	0.77	1.86	11	−0.17	−0.05	0.76	17.25
2	−0.38	−0.37	0.77	3.65	12	−0.16	−0.04	0.76	18.53
3	−0.38	−0.37	0.77	5.38	13	−0.15	−0.03	0.75	19.77
4	−0.35	−0.23	0.77	7.05	14	−0.14	−0.03	0.75	20.97
5	−0.32	−0.20	0.77	8.66	15	−0.14	−0.04	0.75	22.14
6	−0.27	−0.06	0.76	10.21	16	−0.13	−0.06	0.75	23.27
7	−0.24	−0.06	0.76	11.71	17	−0.13	−0.04	0.75	24.37
8	−0.22	−0.06	0.76	13.17	18	−0.12	−0.04	0.75	25.44
9	−0.20	−0.06	0.76	14.57	19	−0.12	−0.03	0.75	26.48
10	−0.19	−0.03	0.76	15.93	20	−0.11	−0.05	0.75	27.49

**Table 5:** Calculated desorption temperatures $T_{D}$ and desorption time τ of the twenty adsorbed H_2_ molecules on Ti–CBQD at different pressures and temperatures.

Pressure (atm)	T_D,min_ (K)	T_D,max_ (K)	T_D,avg_ (K)
1	143.2	499.9	321.5
3	162.9	568.8	365.8
30	229.0	799.6	514.3
100	290.8	998.1	644.5
**Temperature (K)**	**τ** **_min_** **(ns)**	**τ** **_max_**	**τ** **_avg_**
233	0.26	0.28 ms	0.14 ms
298	0.08	4.1 µs	2 µs
358	0.04	0.3 µs	0.2 µs
400	0.03	0.08 µs	0.02 µs

**Table 6:** Comparative overview of reversible hydrogen storage performance in representative nanostructured and solid-state materials reported in the literature.

Material	Metal Decoration	Practical Capacity (wt%)	Ref.
2D C_2_N layer	Li	9	[79]
Graphene	Ti	6–8	[65]
Defected graphene	Li, Mg	8–10	[80]
Carbon nanotubes	Ti	4	[28]
C_60_ fullerene	Li	7	[81]
h-BN	Ti	5.6	[82]
Irida-graphene	Sc	21.6	[62]
R-graphyne-MOF	Li	11.9	[69]
IRMOF-16	Mg	5.8	[83]
MOF-519	-	10	[84]
IRMOF-10	Li	8.3	[85]
COF-1	Sc	5.23	[86]
COF-1	Li	7.7	[87]
CTF-1	Zr	7.1	[88]
AzaCOF	Ti	9.3	[89]
C_8_ quantum dot	Mg	21.7	[90]
C_8_ quantum dot	Ti	3.1	[90]
C_3_B_2_ quantum dot	Ti	20.10	This work

## Data Availability

The original contributions presented in this study are included in the article and Supplementary Materials. Further inquiries can be directed to the corresponding author.

## Supplementary Materials

The following supporting information can be downloaded at: https://www.mdpi.com/article/10.3390/molecules31060960/s1, Figure S1: Optimized geometries for sequential hydrogen adsorption (n = 2–20) on Ti–C3B2 at the ωB97X-3c/vDZP level of theory; Table S1: Calculated adsorption energy and selected geometric parameters of the most stable H2/CBQD configuration obtained using different DFT functionals and DLPNO-CCSD(T) reference method; Table S2: Harmonic vibrational frequencies and IR intensities of pristine CBQD and H2/CBQD; Table S3: Harmonic vibrational frequencies and IR intensities of pristine Ti–CBQD and H2/Ti–CBQD.
