# Supplementary material for: Exceptional Hydrogen Storage Performance of Ti-Decorated C3B2 Quantum Dot: A Comprehensive First-Principles Study
Source: Molecules. 2026 Mar 12;31(6):960. doi: 10.3390/molecules31060960 (PMC13029473; doi:10.3390/molecules31060960)
Supplement: Supplementary file 1 [file molecules-31-00960-s001.zip › molecules-4155976-supplementary.pdf]

Exceptional Hydrogen Storage Performance of Ti-Decorated C3B2 Quantum Dot: A Comprehensive First-Principles Study

Seyfeddine Rahali [1,*], Ridha Ben Said [1], Youghourta Belhocine [2] and Bakheit Mustafa [1,*]

1 Department of Chemistry, College of Science, Qassim University, Buraydah 51452, Saudi Arabia

2 Laboratory of Catalysis, Bioprocess and Environment, Department of Process Engineering, Faculty of Technology, University of 20 August 1955, Skikda 21000, Algeria

* Correspondence: s.rahali@qu.edu.sa (S.R.); b.salih@qu.edu.sa (B.M.)

S1. Benchmark Analysis of DFT Functionals against DLPNO-CCSD(T) for the C_3B_2 Quantum Dot

To assess the reliability of the selected density functional theory (DFT) methods, structural parameters, binding energies, and HOMO–LUMO gaps of the C_3B_2 quantum dot were benchmarked against the high-level DLPNO-CCSD(T)/cc-pVTZ reference method. The results are summarized in Table S1.

Table S1. Calculated adsorption energy and selected geometric parameters of the most stable H_2/CBQD configuration obtained using different DFT functionals and the DLPNO-CCSD(T) reference method.

Method	E_{ads} (eV)	B–H (Å)	B–B (Å)	B–C (Å)	H–H (Å)
**B3LYP-D4/cc-pVTZ**	-0.98	1.332	2.011	1.536	0.839 (0.744)*
**TPSSh/cc-pVTZ**	-0.97	1.338	2.018	1.534	0.835 (0.742)*
**M06-2X/cc-pVTZ**	-0.99	1.337	2.113	1.516	0.828 (0.740)*
**ωB97X-3c**	-0.93	1.365	2.059	1.518	0.836 (0.742)*
**ωB97M-V/QZVP**	-0.95	1.336	2.085	1.531	0.830 (0.738)*
**DLPNO-CCSD(T) /cc-pVTZ**	-0.87	1.326	1.996	1.548	0.839 (0.743)*

S2. Harmonic Vibrational Frequencies

Harmonic vibrational frequency calculations were performed for all optimized structures at the same level of theory as the geometry optimizations in order to confirm their thermodynamic stability. The first six near-zero frequencies correspond to overall translational and rotational motions of the isolated clusters and were therefore excluded from the discussion. All remaining vibrational modes are positive, indicating the absence of imaginary frequencies and confirming that the reported geometries correspond to true local minima on the potential energy surface. The calculated vibrational frequencies, corresponding infrared (IR) intensities and simulated IR spectra are listed in Tables S2 and S3.

Table S2. Harmonic vibrational frequencies and IR intensities of pristine CBQD and H_2/CBQD.

<table>
<tr><th colspan="4">Pristine CBQD</th></tr>
<tr><th>Mode</th><th>Frequency (cm^{-1})</th><th>IR Intensity (km mol^{-1})</th><th>Simulated IR spectra</th></tr>
<tr><td>6</td><td>435.40</td><td>0.26</td><td rowspan="9">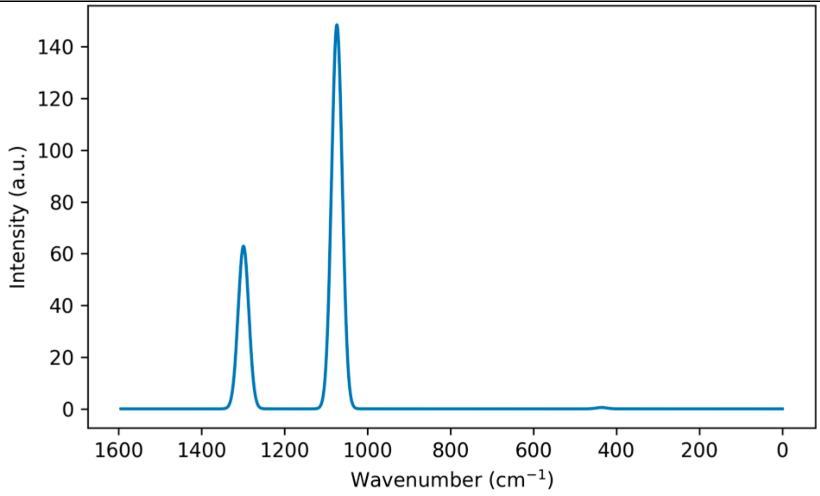
</td></tr>
<tr><td>7</td><td>435.47</td><td>0.26</td></tr>
<tr><td>8</td><td>452.93</td><td>0.00</td></tr>
<tr><td>9</td><td>937.95</td><td>0.00</td></tr>
<tr><td>10</td><td>938.43</td><td>0.00</td></tr>
<tr><td>11</td><td>1073.17</td><td>73.94</td></tr>
<tr><td>12</td><td>1073.91</td><td>74.60</td></tr>
<tr><td>13</td><td>1298.71</td><td>62.89</td></tr>
<tr><td>14</td><td>1394.19</td><td>0.00</td></tr>
<tr><th colspan="4">H2/CBQD</th></tr>
<tr><th>Mode</th><th>Frequency (cm^{-1})</th><th>IR Intensity (km mol^{-1})</th><th>Simulated IR spectra</th></tr>
<tr><td>6</td><td>145.46</td><td>0.83</td><td rowspan="15">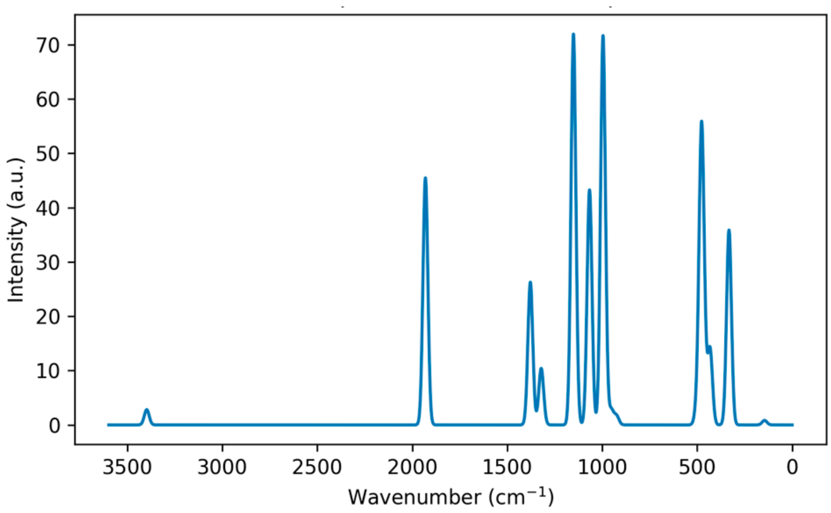
</td></tr>
<tr><td>7</td><td>332.49</td><td>35.87</td></tr>
<tr><td>8</td><td>432.20</td><td>13.81</td></tr>
<tr><td>9</td><td>462.57</td><td>7.69</td></tr>
<tr><td>10</td><td>477.55</td><td>51.14</td></tr>
<tr><td>11</td><td>499.28</td><td>3.36</td></tr>
<tr><td>12</td><td>924.55</td><td>1.67</td></tr>
<tr><td>13</td><td>953.14</td><td>2.78</td></tr>
<tr><td>14</td><td>995.77</td><td>71.66</td></tr>
<tr><td>15</td><td>1066.73</td><td>43.26</td></tr>
<tr><td>16</td><td>1151.36</td><td>71.94</td></tr>
<tr><td>17</td><td>1321.07</td><td>10.40</td></tr>
<tr><td>18</td><td>1378.29</td><td>26.26</td></tr>
<tr><td>19</td><td>1930.68</td><td>45.48</td></tr>
<tr><td>20</td><td>3397.80</td><td>2.81</td></tr>
</table>

Table S3. Harmonic vibrational frequencies and IR intensities of pristine Ti-CBQD and H_2/Ti-CBQD.

Ti-CBQD

Mode	Frequency (cm^{-1})	IR Intensity (km mol^{-1})	Infrared spectra
6	219.29	5.82	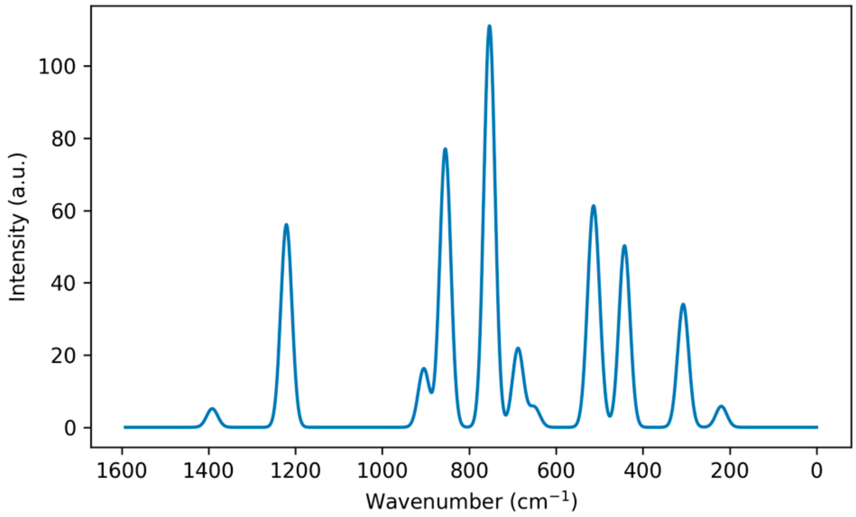
7	307.14	34.06	
8	442.19	50.27	
9	506.69	24.52	
10	517.04	41.65	
11	649.76	5.53	
12	687.46	21.83	
13	753.09	111.15	
14	854.84	77.08	
15	904.35	16.24	
16	1220.66	56.12	
17	1391.42	5.16	

H2/Ti-CBQD

Mode	Frequency (cm^{-1})	IR Intensity (km mol^{-1})	Infrared spectra
6	167.04	1.90	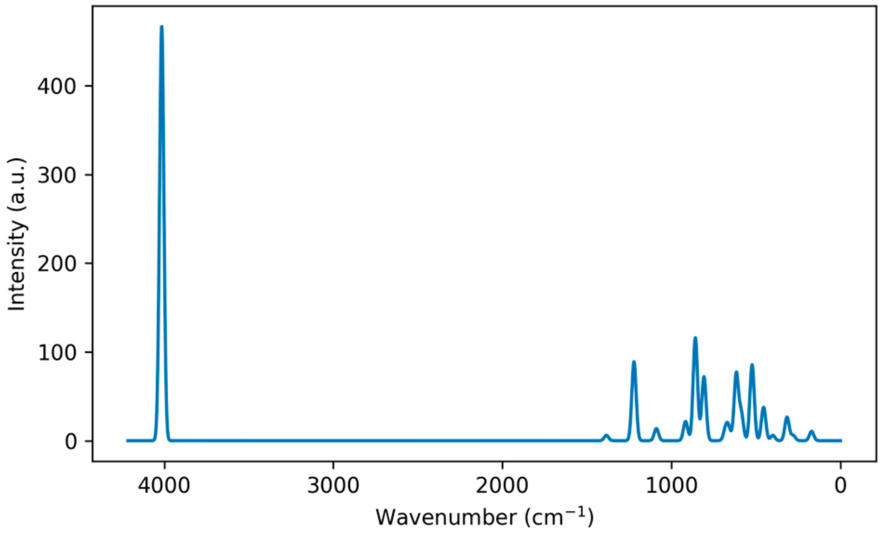
7	171.44	8.72	
8	279.42	5.91	
9	316.18	26.51	
10	399.94	6.12	
11	454.59	37.71	
12	522.62	85.54	
13	586.74	30.17	
14	616.17	75.15	
15	661.93	13.13	
16	679.10	12.76	
17	807.38	72.13	
18	858.34	115.89	
19	916.05	21.70	
20	1088.89	13.67	
21	1221.44	88.96	
22	1384.56	6.17	
23	4015.12	466.71	

S3. Sequential Hydrogen Uptake on Ti–C_3B_2 Quantum Dot

The sequential adsorption of hydrogen molecules on Ti–C_3B_2 was investigated by progressively adding H_2 molecules and fully optimizing each configuration at the ωB97X-3c/vDZP level. For each loading step (n = 1–20), multiple initial orientations and positions of H_2 were considered to ensure convergence toward the lowest-energy structure. The first five H_2 molecules coordinate directly to the Ti center through η^2-H_2 (Kubas-type) interactions, characterized by moderate Ti–H_2 distances and slight H–H bond elongation. Beyond this coordination limit, additional hydrogen molecules are stabilized over the quantum-dot surface through weaker cooperative interactions involving polarization effects and intermolecular stabilization among adsorbed H_2 molecules. The optimized structures shown in Figure S1 illustrate the gradual increase in hydrogen coverage and confirm that the Ti center acts as the primary adsorption site during the initial uptake stage, while higher loadings are accommodated by surface-mediated interactions without the structural collapse of the C_3B_2 framework. These results support the distinction between the local Ti coordination limit (five H_2 molecules) and the overall hydrogen uptake capacity of the Ti–C_3B_2 system.

No spontaneous H_2 dissociation or Ti migration was observed during sequential loading, and the C_3B_2 framework preserves its structural integrity throughout the adsorption sequence, confirming the robustness of the decorated quantum dot.

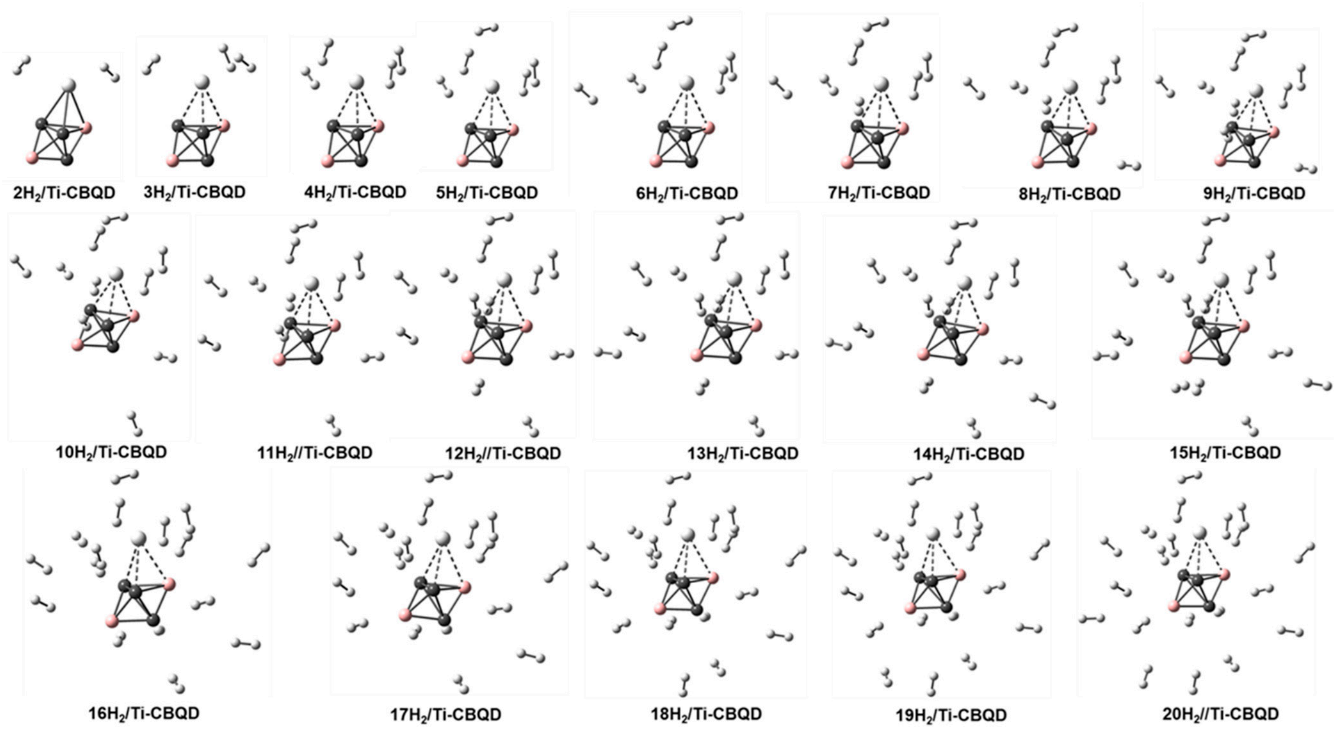

Figure S1. Optimized geometries for sequential hydrogen adsorption (n = 2–20) on Ti–C_3B_2 at the ωB97X-3c/vDZP level of theory

S4. Cartesian coordinates (in angstroms) performed at the ωB97X-3c/vDZP level of theory

Pristine C_3B_2

**C**	**7.66240000092514**	**7.96955584111878**	**6.68779982780973**
**C**	**7.66240000139969**	**6.65505510674166**	**8.10935253743592**

C	7.66240000132015	6.08064999670354	6.25975634066563
B	8.64123674245212	6.90166952731779	7.01874564724755
B	6.68356325390287	6.90166952811822	7.01874564684115

Pristine H2/C_3B_2

C	8.06427340143986	7.70294770514898	6.48513389401733
C	7.97594357367249	6.40386474836980	7.90619842199381
C	7.08480215815939	6.11905300424960	6.28252079477532
B	8.58153967351730	6.27608621647297	6.51994711550265
B	6.91197085529781	7.21541190951482	7.27515652168699
H	9.84200373515031	5.83772465913692	6.19340798607137
H	9.28616845276261	5.39056878710677	5.75658434595235

Ti-C_3B_2

C	7.92841892503905	7.58758356422557	6.76557184277459
C	7.96100762640015	6.50293614048495	7.94639512269596
C	7.58429695037665	6.01288924518773	6.37279151175351
B	9.01188051869136	6.39550134841712	6.81320918228917
B	6.49285333814554	6.68332895203575	7.03289187417256
Ti	6.93987139134718	8.20140175964881	8.33089902631413

H_2/C_3B_2-Ti

C	7.34818024250200	7.58551831994612	6.94280897917233
C	7.62779776674059	6.02055477843626	6.77201423173911
C	6.06140124784414	6.63738483232269	6.52967114254977
B	7.20669383678929	6.83114362108032	5.51807726239554
B	6.42959717489136	6.51995931391923	7.92025978933835
Ti	8.36696001907077	6.90043821466201	8.44427544929416
H	8.61177174116758	8.63324503769412	9.61706891411734
H	7.89556826099422	8.39227170193920	9.77120296139335

$20H_2/C_3B_2$-Ti

C	6.75135819953959	7.10115452380875	6.55984988730555
C	7.94303706884538	6.66941803208823	5.58560069051437
C	6.79946095972535	5.52404763962804	6.00146061727213
B	6.46631237385879	6.72809200930228	5.06637390651915
B	7.74448320470273	5.83690892240670	7.03231643206502
Ti	8.59376578157619	7.72478598253307	7.22207437714679
H	10.82652777772584	7.75932293607310	6.42016067893710
H	10.44372361199036	7.35999531820815	5.90218569957419
H	7.67497802098063	8.57652231549011	9.23862880456223
H	7.09073029138266	8.25407879917408	8.88051534313806
H	9.74968092472845	6.89808092966280	8.80567840591165
H	9.26780532401499	6.31658365249166	8.52042265535295
H	8.37849625268280	9.48148944627928	5.70701997467857
H	7.76678588245527	9.60469564082068	6.13681233101935
H	10.04354949975632	9.32780240922752	8.35724064905015
H	9.71535291944505	9.79633815649671	7.87033079207846
H	9.64689819980826	8.89530454089409	3.25814681083728
H	9.22916504356489	8.33100845149990	3.50905983328991
H	10.61180532010619	4.57805420586782	6.83999706454706
H	11.26468237996848	4.54383414292377	7.19678981836466
H	5.02666722465755	6.40160389710142	9.62056952562627
H	5.22306102574205	6.40811952823749	8.90173636554226
H	5.77216361062059	10.70782065433632	7.82648747132281
H	5.57035515670743	10.07009829127845	7.49931266045556

H	3.34730476380440	4.93034958874218	6.70857895624296
H	4.01570038825992	4.95883006887585	6.38134002839247
H	5.38170475578199	10.23143140133403	4.53229909671261
H	5.42382423315626	9.49977285654907	4.66641445073394
H	3.91769208505056	8.01329066496123	6.72144856304919
H	3.27508881167414	8.36218483501915	6.86572037891886
H	10.37621139596080	5.69310964542008	4.04186002745000
H	11.10846460988918	5.69431231400465	3.90491985720682
H	5.93661007053139	2.69926322056108	8.47683768535901
H	6.02327503540263	3.20820437964160	7.94048704134783
H	9.12548288511494	2.97842859012322	4.82884973546034
H	8.56845226441270	3.43673440558831	5.01324344086578
H	9.13043862756287	3.76398470636811	9.99675711805213
H	8.65437628087224	3.87176499201358	9.43507026612556
H	7.93397625279484	5.44498179422298	2.76774714005687
H	8.06385284026817	5.15374940294391	2.09467187896249
H	5.18661729704499	3.28710403461900	3.46104906655845
H	5.46187973429183	3.75553589199817	3.97044302251158
H	3.84461960970117	6.66661175733670	3.58419332713657
H	3.16429416144625	6.64763483283085	3.28164031233417
H	6.51370973669175	8.42299124872207	1.65182109442413
H	6.48330072569889	8.02381036229167	2.27989000698493
